# Role of miR-200c in Myogenic Differentiation Impairment via p66Shc: Implication in Skeletal Muscle Regeneration of Dystrophic *mdx* Mice

**DOI:** 10.1155/2018/4814696

**Published:** 2018-02-13

**Authors:** Marco D'Agostino, Alessio Torcinaro, Luca Madaro, Lorenza Marchetti, Sara Sileno, Sara Beji, Chiara Salis, Daisy Proietti, Giulia Imeneo, Maurizio C. Capogrossi, Francesca De Santa, Alessandra Magenta

**Affiliations:** ^1^Department of Experimental Medicine, Sapienza University of Rome, Viale Regina Elena 324, 00161 Rome, Italy; ^2^Department of Biology and Biotechnology “Charles Darwin”, Sapienza University of Rome, Roma, Italy; ^3^Institute of Cell Biology and Neurobiology (IBCN), Italian National Research Council (CNR), 00143 Rome, Italy; ^4^Fondazione Santa Lucia IRCCS, 00143 Rome, Italy; ^5^Vascular Pathology Laboratory, Instituto Dermopatico dell'Immacolata-IRCCS, FLMM, Via dei Monti di Creta 104, 00167 Rome, Italy; ^6^Department of Cardiology, Ochsner Medical Center, 1514 Jefferson Hwy., New Orleans, LA 70121, USA

## Abstract

Duchenne muscular dystrophy (DMD) is a genetic disease associated with mutations of Dystrophin gene that regulate myofiber integrity and muscle degeneration, characterized by oxidative stress increase. We previously published that reactive oxygen species (ROS) induce miR-200c that is responsible for apoptosis and senescence. Moreover, we demonstrated that miR-200c increases ROS production and phosphorylates p66Shc in Ser-36. p66Shc plays an important role in muscle differentiation; we previously showed that p66Shc^−/−^ muscle satellite cells display lower oxidative stress levels and higher proliferation rate and differentiated faster than wild-type (*wt*) cells. Moreover, myogenic conversion, induced by MyoD overexpression, is more efficient in p66Shc^−/−^ fibroblasts compared to *wt* cells. Herein, we report that miR-200c overexpression in cultured myoblasts impairs skeletal muscle differentiation. Further, its overexpression in differentiated myotubes decreases differentiation indexes. Moreover, anti-miR-200c treatment ameliorates myogenic differentiation. In keeping, we found that miR-200c and p66Shc Ser-36 phosphorylation increase in *mdx* muscles. In conclusion, miR-200c inhibits muscle differentiation, whereas its inhibition ameliorates differentiation and its expression levels are increased in *mdx* mice and in differentiated human myoblasts of DMD. Therefore, miR-200c might be responsible for muscle wasting and myotube loss, most probably via a p66Shc-dependent mechanism in a pathological disease such as DMD.

## 1. Introduction

We previously showed that oxidative stress inhibits myogenic differentiation [1] and in a model of oxidative stress such as acute hind limb ischemia, it was demonstrated that reactive oxygen species (ROS) production plays a causal role in tissue damage, leading to cell death by both apoptosis and necrosis [2].

p66Shc adaptor protein is a redox enzyme implicated in mitochondrial ROS generation and translation of oxidative signals [3]. Under physiological conditions, the phosphorylation of Tyr residues of p66Shc by growth factors mediates the signal transduction to the nucleus, inhibiting the Ras signaling pathway, while phosphorylation of the Ser-36 site is crucial for oxidative stress response [4]. p66Shc once phosphorylated in Ser-36 enhances ROS production by using three different mechanisms restricted in the nucleus, the plasma membrane, and the mitochondria [4].

In keeping, our previous results demonstrated that p66Shc inhibits myogenic differentiation and p66Shc deletion enhances skeletal muscle regeneration after ischemia [1].

MicroRNAs (miRNAs) are 21–23 nucleotide RNA molecules that regulate stability or translational efficiency of target messenger RNAs [5]. miRNAs control a wide range of cell functions and have been associated with inflammation, oxidative stress, and differentiation [6–8].

We previously showed that the miR-200 family is upregulated upon oxidative stress in different cells, such as endothelial cells, human fibroblasts, murine myoblasts, and myotubes [9]. This miRNA family consists of five members (miR-200c, miR-141, miR-200a, miR-200b, and miR-429). We demonstrated that miR-200c is the most upregulated family member and is responsible for apoptosis and senescence by targeting zinc finger E-box-binding homeobox 1 (ZEB1) protein [9]. We also demonstrated that miR-200c is induced following acute hind limb ischemia in skeletal muscles and this induction was oxidative stress dependent, since in p66Shc^−/−^ mice, which exhibit less oxidative stress than wild-type (*wt*) mice [1], miR-200c increase is significantly attenuated [9].

In a recent publication, we demonstrated that miR-200c increased ROS production and induced p66Shc protein phosphorylation in Ser-36; this mechanism upregulated ROS and inhibited FOXO1 transcription of ROS scavengers, reinforcing this molecular circuitry [10].

Moreover, we showed that anti-miR-200c treatment in hind limb ischemia in mice rescued the decrease of miR-200c protein targets and improved limb perfusion [10].

Herein, we wanted to dissect the role of miR-200c in muscle differentiation and to comprehend whether miR-200c levels were modulated in muscle pathological diseases associated with oxidative stress increase, such as Duchenne muscular dystrophy (DMD) [11, 12].

In keeping with this hypothesis, in the paper of Greco et al., an interesting link between ischemia-, *mdx*-, and DMD-modulated miRNAs associated with apoptosis/myonecrosis was demonstrated [13]. Interestingly, in adductor muscles of *mdx*, a miR-200c upregulation was found in a miRNA screening, although not significantly [13].

Muscular dystrophies are a heterogenous group of genetic disorders characterized by muscle degeneration and associated with mutations of genes that regulate myofiber integrity [14]. The most common dystrophy is the DMD, a lethal X-linked genetic disease characterized by severe muscle degeneration, caused by deficiency of dystrophin, a critical component of the dystrophin glycoprotein complex (DGC), acting as a link between cytoskeleton and extracellular matrix both in skeletal and cardiac muscles [15, 16]. The *mdx* mice strain represents the most used animal model to study DMD [17].

Dystrophic muscles undergo continuous cycles of degeneration and regeneration. Satellite cells (SC), the skeletal muscle stem cells, exit from quiescence and undergo the proliferation phase followed by activation of skeletal muscle differentiation program or return to quiescence to maintain the stem cell pool. Although SC compensate for muscle fiber loss in the early stages of dystrophy leading to muscle compensatory regeneration, eventually, these progenitors become exhausted [18]. As a result, muscles are characterized by necrosis and inflammation culminating in extracellular matrix and fat deposition. Consequently, fibrous and fatty connective tissue overtakes the functional myofibers [15, 19].

Recent papers highlighted new cellular and molecular mechanisms contributing to SC dysfunction in dystrophic muscle. Specifically, SC hold an intrinsic cell dysfunction affecting their polarity and asymmetric division [20]. Moreover, SC can undergo mesenchymal fibrogenic conversion, mediated by TGFbeta signaling, compromising their physiological muscle regenerative functions [21, 22].

The pathology of DMD appears to be exacerbated by oxidative stress, and ROS increase plays a pivotal role in the necrosis of skeletal muscles in DMD and in dystrophic *mdx* mouse [11]. Moreover, since contractile (myofibrillar) proteins such as myosin, actin, troponin, and tropomyosin containing thiol side chains are sensitive to oxidation, these modifications may alter excitation/contraction coupling and cross-bridge cycling, modulating muscle contraction. As a consequence, excessive oxidative stress that occurs in DMD provokes muscle weakness and wasting [11].

The results of the present work show that miR-200c impairs muscle differentiation, whereas miR-200c inhibition ameliorates differentiation; moreover, both miR-200c expression levels and p66Shc phosphorylation in Ser-36 increase in *mdx* mice. Moreover, miR-200c increases also in differentiated human myoblasts of DMD. Therefore, we hypothesized a miR-200c role in muscle wasting and myotube loss via a p66Shc-dependent mechanism in DMD.

## 2. Materials and Methods

### 2.1. Cell Line, Culture Conditions, and Transfections

C2.12 (C2C12), a subclone of the C2 mouse myoblast cell line, was obtained from M Buckingham. C2C12 were cultured in growth medium ((GM) DMEM-GlutaMAX complemented with penicillin/streptomycin and 20% FBS). Myogenic differentiation was induced by shifting the cells in differentiation medium ((DM) DMEM-GlutaMAX complemented with penicillin/streptomycin and 2% FBS).

Human myoblasts were derived from muscle biopsies of healthy donors or DMD patients. Human myoblasts were cultured in growth medium (GM) (DMEM-GlutaMAX complemented with penicillin/streptomycin and 20% FBS). Myogenic differentiation was induced by shifting the cells in differentiation medium (DM) (DMEM-GlutaMAX complemented with penicillin/streptomycin, 5% horse serum, and insulin 100 *μ*g/ml).

Transfections were carried out by using Lipofectamine 3000 reagent (Invitrogen) according to the manufacturer's instructions. Cells were seeded at 10^5^ per well in six-well dishes and transfected 18 hours (h) later. The amount of plasmid used in transfection assay is indicated in the figure legend.

### 2.2. Drug Treatments

H_2_O_2_ (30% (wt/wt) solution; Sigma) was administered to the cells as a 100 mM solution in phosphate-buffered saline (PBS).

### 2.3. Plasmid Constructs

p66Shc-Ser-36 to Ala mutant was generated using QuickChange Site-Directed Mutagenesis Kit (Stratagene) starting from p66Shc pBABE vector. plko.1-miR-200c and plko.1-anti-miR-200c constructs were described previously [9, 10].

### 2.4. miRNA Overexpression and Inhibition

Stable expression of miR-200c, anti-miR-200c, or miR-scramble in C2C12 cells was generated by viral infection using lentiviral supernatants. These viruses were produced as previously described [23]. In summary, cells were infected with lentiviral virus for 2 h and then were recovered in complete fresh medium for 24 h. Afterwards, infected cells were selected by puromycin-containing medium (Sigma) for 72 h. miR-200c overexpression was controlled by quantitative real-time PCR (RT-qPCR) (see methods below).

### 2.5. Immunofluorescence of Cultured Cells

C2C12 in culture were fixed in 4% paraformaldehyde in PBS for 10 minutes at room temperature, incubated with glycine 50 mM in PBS for 10 minutes at room temperature to quench paraformaldehyde, and permeabilized with 0.1% Triton-X in PBS for 10 min at room temperature. Then, cells were blocked with 4% IgG-free bovine serum albumin (BSA) in PBS for 30 minutes. Cells were immune labelled with the antibody against myosin heavy chain (MyHC) (MF20 Hybridoma bank) in 4% BSA overnight at +4°C. Donkey anti-mouse IgG conjugated to Alexa Fluor 488 (Jackson ImmunoResearch #715-545-150) were used to detect the signal. Nuclei were counterstained with DAPI (Sigma D9542). Phase contrast images of C2C12 cells were acquired with Leica microscope (DM-IRB). Immunofluorescence images were acquired with confocal laser scanning microscopy system Zeiss Axiovert 200M or fluorescence microscope Nikon Eclipse TE-2000E. Counts were performed with ImageJ software.

Differentiation index calculations are as follows:

- *Differentiation index* was measured as the percentage of all MyHC^+^ cells, both mononucleated and multinucleated cells.
- *Fusion index* was measured as the percentage of multinucleated MyHC^+^ cells (≥2 nuclei).
- *Nuclei per myotube* were calculated as the mean of the number of nuclei within myotubes.

### 2.6. Animal Model

*Mdx* mice (C57BL10J DMD*^mdx^*) and wild-type (*wt*) mice (C57BL10J) were purchased from Charles River. All mice handling procedures were approved by the internal Animal Research Ethical Committee according to the Italian Ministry of Health and complied with the NIH Guide for the Care and Use of Laboratory Animals. All the procedures were carried out in accordance with the promise of the three Rs (replacement, reduction, and refinement). The animals were housed in cages with environmental enrichment in order to reduce pain and stress and increase animal welfare. The animals were sacrificed, and hind limb muscles were directly frozen in liquid nitrogen and stored at −80°C.

### 2.7. RNA Isolation and qPCR Analysis

Hind limb muscles from *mdx* mice were homogenized by a handheld rotor-stator homogenizer (TissueRuptor—Qiagen) in TRIzol reagent (Invitrogen). RNA was extracted following manufacturer's protocol (TRIzol—Invitrogen).

Hind limb muscles were isolated from 3 different animals for each strain and age described in the figures, and RNA was isolated and quantified by NanoDrop (Thermo Scientific 2000C).

miRNA levels were analyzed using the TaqMan RT-qPCR and quantified with the ABI Prism 7000 SDS (Applied Biosystems). miR-200c levels were normalized to U6 small RNA expression as previously reported [24, 25].

Primers for miR-200c, U6, and reagents for reverse transcriptase and RT-qPCRs were all obtained from Applied Biosystems.

### 2.8. Protein Isolation and Western Blot Analysis

C2C12 cells were lysed in a buffer containing 100 mM Tris (pH 6.8), 20% glycerol, and 4% sodium dodecyl sulfate (SDS). Amounts of protein were determined by bicinchoninic acid protein assay kit (Pierce, Rockford, IL). Then dithiothreitol (DTT) (200 mM) was added and lysates were boiled for 5 min.

Hind limb muscles from *mdx* mice were homogenized by a handheld rotor-stator homogenizer (TissueRuptor—Qiagen; 5–10 seconds, 4 times at +4°C) in protein extraction buffer containing 50 mM Tris-HCl pH 7.5, 0.6 M sucrose, 50% glycerol, 1% Triton, and 50 mM NaCl supplemented with protease (1 mM PMSF, 5 *μ*g/ml aprotinin, 5 *μ*g/ml leupeptin, and 5 *μ*g/ml pepstatin) and phosphatase inhibitors (10 mM NaF, 5 mM *β*-glycerophosphate, and 1 mM Na-orthovanadate). Lysates were also sonicated (5 seconds, 2 times), incubated on a tube rotator at +4°C for 30 minutes, and cleared of insoluble debris by centrifugation at 13000 rpm for 20 minutes at +4°C and the supernatants were stored at −80°C. Protein concentrations were determined by Bradford assay.

For Western blot analysis, proteins were extracted from gastrocnemius and quadriceps muscles of *wt* and *mdx* animals (3 *wt* mice and 3 *mdx* mice of 4 weeks and 36 weeks, resp.). Proteins were separated on denaturing SDS-polyacrylamide gels, transferred to the nitrocellulose membrane by standard procedures, and blotted with the following primary antibodies: ZEB1 (H-102), myosin heavy chain MyHC (MF20 mouse hybridoma), MyoD (MoAb 5.8A, Dako), myogenin (IF5D mouse hybridoma), p66Shc (Transduction Laboratories), p66Shc-phospho-Ser-36 (Abcam 6E10), tubulin (Oncogene Research Products Ab-1), and GAPDH (Calbiochem CB1001). The antibody binding was revealed by horseradish peroxidase-conjugated secondary antibodies followed by chemiluminescence detection (ECL, Pierce).

Immunoprecipitations were performed as previously described [26]. Cells were resuspended in lysis buffer containing 50 mM HEPES (pH 7.5), 250 mM NaCl, 1 mM DTT, 0.1% Tween 20, 10% glycerol, 5 mM CaCl2, 1 mM phenylmethylsulfonyl fluoride (PMSF), 10 mM Na_3_VO_4_, 50 mM NaF, and protease inhibitors (complete EDTA-free protease inhibitor mixture tablets; Roche Applied Sciences). Immunoprecipitations were performed for 2 to 3 h at 4°C with protein A/G agarose and 1 *μ*g of relevant antibodies. Immune complexes were resuspended in 2x Laemmli buffer, separated by SDS-polyacrylamide gel electrophoresis (PAGE), and immunoblotted with relevant antibodies.

### 2.9. Statistical Analysis

The number of samples or independent experiments and the definition of reported values are indicated in the figure legends as mean ± standard error of the mean (SEM). Statistical analyses were performed using the GraphPad Prism software (Version 5.0). Statistical significance was assessed by unpaired Student's *t*-test or ANOVA. *P* value < 0.05 was considered as statistically significant.

## 3. Results

### 3.1. miR-200c Overexpression Inhibits Myogenic Differentiation

We previously showed that oxidative stress inhibits muscle differentiation [1]. We also demonstrated that miR-200c is highly induced upon H_2_O_2_ treatment in C2C12 in both myoblasts and differentiated myotubes [9]. Therefore, we asked whether miR-200c modulation had an effect on myogenic differentiation.

**Figure 1:**
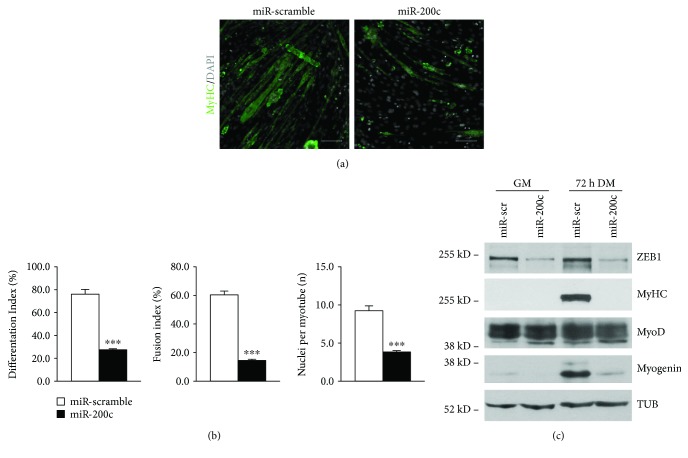
miR-200c overexpression in myoblasts inhibits skeletal muscle differentiation *in vitro*. C2C12 myoblasts were infected either with a lentivirus encoding miR-200c or with a control virus. After selection with puromycin, cells were plated and shifted to differentiation medium for 3 days. (a) Representative images of anti-MyHC staining (green). Nuclei were counterstained with DAPI (grey). Immunofluorescence with anti-MyHC antibody showed a decrease in myotubes in miR-200c-overexpressing cells compared to control. Scale bar: 200 *μ*m. (b) Bar graphs representing differentiation index (percentage of MyHC-positive cells), fusion index (percentage of nuclei within a myotube), and number of nuclei per myotube. miR-200c overexpression decreased all these parameters (*n* = 3 independent experiments; ^∗∗∗^*p* < 0.001). (c) A representative Western blot using ZEB1, MyHC, myogenin, and antibodies showed that protein levels decreased upon miR-200c overexpression and MyoD expression was not affected. *α*-Tubulin (TUB) was used as loading control.

To this aim, we overexpressed miR-200c in C2C12 myoblasts; then, we shifted the cells to differentiation medium (DM). We found that miR-200c inhibited myotube formation as assessed by MyHC immunofluorescence staining (Figure 1(a)). In addition, a decrease of three muscle differentiation parameters was also observed, specifically differentiation index (percentage of both myotubes and MyHC-positive cells), fusion index (percentage of nuclei within a myotube), and number of nuclei within myotubes (Figure 1(b)). We then analyzed myogenic differentiation by Western blot analysis, and we observed that ZEB1, MyHC, and myogenin proteins were all downregulated upon miR-200c overexpression, whereas MyoD was not affected (Figure 1(c)).

**Figure 2:**
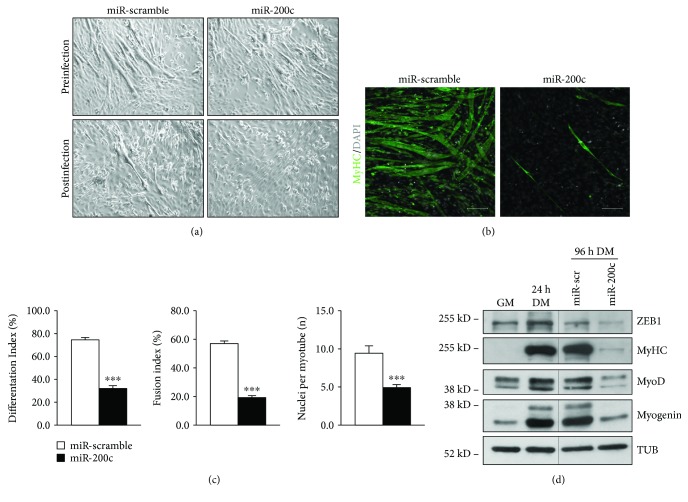
miR-200c overexpression in myotubes inhibits skeletal muscle differentiation *in vitro*. C2C12 myoblasts were shifted to differentiation medium for 24 hrs; then, cells were infected either with a lentivirus encoding miR-200c or with a control virus. Afterwards, cells were selected with puromycin in differentiation medium for 3 days. (a) Representative-phase contrast images of C2C12 myoblasts prior to infection (upper panels) and after infection (lower panels). (b) Representative images of anti-MyHC staining (green). Nuclei were counterstained with DAPI (grey). Scale bar: 200 *μ*m. (c) Bar graphs representing differentiation index, fusion index, and number of nuclei per myotube. miR-200c overexpression decreased all these parameters (*n* = 3 independent experiments; ^∗∗∗^*p* < 0.001). (d) A representative Western blot using ZEB1, MyHC, myogenin, and MyoD antibodies showed that protein levels decreased upon miR-200c overexpression. *α*-Tubulin (TUB) was used as loading control.

miR-200c overexpression was also performed in C2C12 after 24 hrs of myogenic differentiation. As shown in phase contrast images of Figure 2(a), we started from cells with a similar degree of differentiation prior to infection (upper panels); we found that miR-200c overexpression decreased the myotube number (Figure 2(a) lower panels), assessed also by MyHC immunofluorescence staining (Figure 2(b)). Moreover, a decrease in differentiation index, fusion index, and number of nuclei within myotubes was also observed in differentiated miR-200c-overexpressing cells (Figure 2(c)).

We then analyzed myogenic differentiation by Western blot, and we observed that ZEB1, MyHC, myogenin, and MyoD proteins were all downregulated upon miR-200c overexpression (Figure 2(d)).

All these results suggested a role for miR-200c in myogenic differentiation inhibition and in myotube loss.

### 3.2. miR-200c Inhibition Enhances Myogenic Differentiation

**Figure 3:**
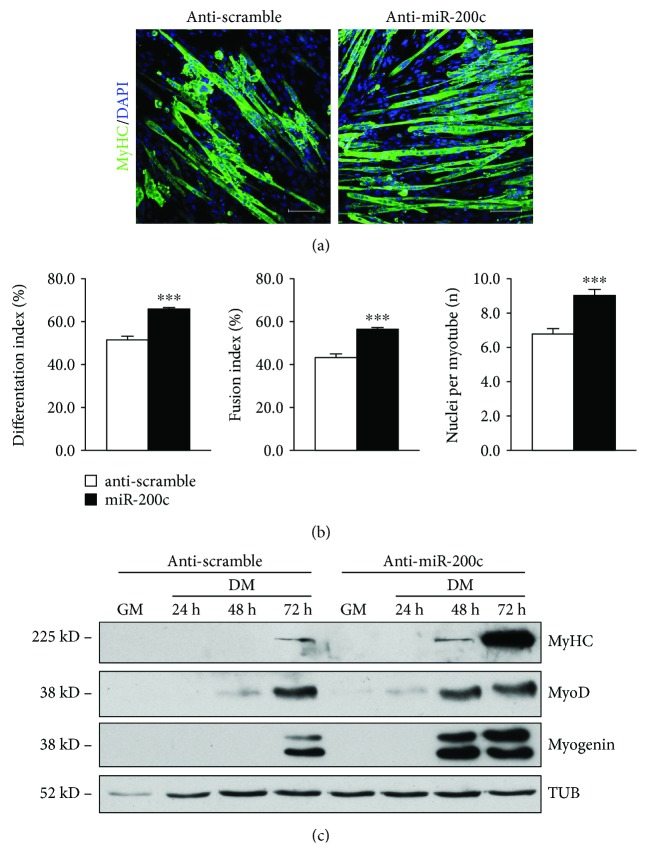
Anti-miR-200c treatment enhances skeletal muscle differentiation *in vitro*. C2C12 myoblasts were infected either with a lentivirus encoding anti-miR-200c or with a control virus. After selection with puromycin, cells were plated and shifted to differentiation medium for the times indicated in the figure. (a) Representative images of anti-MyHC staining (green). Nuclei were counterstained with DAPI (blue). Immunofluorescence with anti-MyHC antibody showed an increase in myotubes in anti-miR-200c-overexpressing cells compared to control at 3 days in DM. Scale bar: 100 *μ*m. (b) Bar graphs representing differentiation index, fusion index, and number of nuclei per myotube. Anti-miR-200c overexpression increased all these parameters (*n* = 3 independent experiments; ^∗∗∗^*p* < 0.001). (c) A representative Western blot using MyHC, myogenin, and MyoD antibodies showed that all these protein levels increased upon anti-miR-200c overexpression. *α*-Tubulin (TUB) was used as loading control.

We then asked whether anti-miR-200c treatment was able to ameliorate myogenic differentiation. Therefore, we transduced C2C12 cells with anti-miR-200c lentiviral particles and we shifted cells to DM for increasing period of times. We found that anti-miR-200c increased myotube formation as assessed by MyHC immunofluorescence staining (Figure 3(a)). In addition, an increase of three muscle differentiation parameters was also observed, specifically differentiation index, fusion index, and number of nuclei within myotubes (Figure 3(b)). We analyzed myogenic differentiation by Western blot, and we found that MyHC, myogenin, and MyoD proteins were increased at 48 h and 72 h of DM upon anti-miR-200c expression at higher levels compared to anti-scramble-treated C2C12 (Figure 3(c)).

### 3.3. miR-200c Increased p66Shc Phosphorylation in Ser-36 in C2C12 Myoblasts

**Figure 4:**
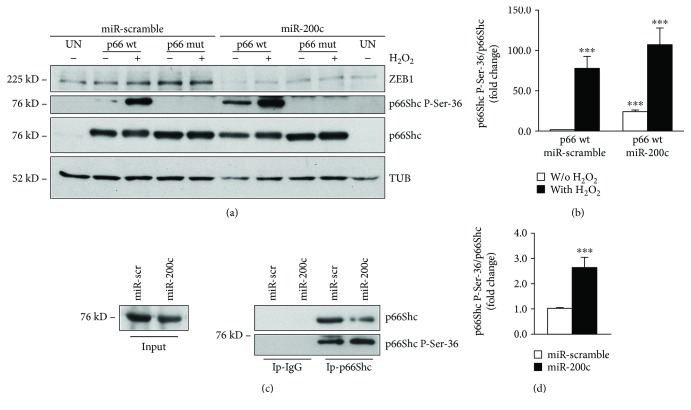
miR-200c overexpression induces p66Shc phosphorylation in Ser-36. C2C12 myoblasts were infected either with a lentivirus encoding miR-200c or with a control virus. After selection with puromycin, cells were transfected with 1 *μ*g of p66*wt* or a mutated version in which Ser-36 was substituted with Ala that was no longer phosphorylable (p66mut). (a) A representative Western blot using p66Shc-Ser-36 antibody showed that p66*wt* phosphorylation was higher in miR-200c-overexpressing cells compared to scramble control both without and with H_2_O_2_ treatment. Phosphorylation of p66mut was not present, as expected, in any condition. *α*-Tubulin (TUB) was used as loading control. (b) Bar graph showing the quantification of p66Shc phosphorylation in Ser-36 versus p66*wt* protein levels of C2C12-overexpressing miR-200c compared with control cells (*n* = 3; ^∗∗∗^*p* < 0.001). (c) C2C12 myoblasts were infected either with a lentivirus encoding miR-200c or with a control virus. After selection with puromycin, cells were immunoprecipitated (Ip) with either an anti-p66 antibody or an irrelevant isotypic antibody (negative control). Western blotting with a p66Shc-phospho-Ser-36 antibody revealed that p66Shc was more phosphorylated in Ser-36 in miR-200c IP-p66 than in scramble control cells. The efficiency of immunoprecipitation was assessed with an anti-p66 antibody. One-twentieth of the immunoprecipitated whole-cell extract (input) was loaded as a reference. (d) Bar graph showing the quantification of p66Shc phosphorylation in Ser-36 versus p66 total protein levels of C2C12-overexpressing miR-200c compared with scramble control cells (*n* = 3; ^∗∗∗^*p* < 0.001).

We previously showed that, in endothelial cells, miR-200c induces p66Shc phosphorylation in Ser-36, a phosphorylation known to be elicited by oxidative stress [10]. We therefore asked whether miR-200c phosphorylated p66Shc in this residue, also in C2C12 myoblasts. To this aim, we transduced C2C12 with miR-200c and scramble control and then transfected the cells with a p66Shc *wt* cells or a mutated p66 (p66mut) plasmid in which Ser-36 was replaced with Ala, that is not phosphorylable. We treated cells with or without 400 *μ*M H_2_O_2_ for 5 minutes, and we found that Ser-36 phosphorylation increased, as expected, upon H_2_O_2_ treatment in p66*wt*-transfected cells, but not in the p66mut-transfected ones (Figures 4(a) and 4(b)). Moreover, in C2C12-overexpressing miR-200c, p66*wt* was phosphorylated also in basal conditions, that is, without H_2_O_2_, and the phosphorylation in Ser-36 increased even further upon H_2_O_2_ treatment (Figures 4(a) and 4(b)).

Further, we aimed at establishing whether endogenous p66 was phosphorylated in Ser-36 by miR-200c. Unfortunately, we failed to visualize phosphorylation by Western blot analysis; therefore, we immunoprecipitated p66Shc, to enhance the signal of Ser-36 phosphorylation.

As shown in Figure 4c, we found an increase in Ser-36 phosphorylation in the immunoprecipitates of p66 in miR-200c-overexpressing C2C12 compared to scramble control (Figures 4(c) and 4(d)).

Taken together, these results indicate that miR-200c enhances p66Shc phosphorylation in Ser-36 as well as in C2C12 myoblasts, supporting its role in oxidative stress production [10].

### 3.4. miR-200c and p66Shc Phosphorylation in Ser-36 Increase in Skeletal Muscles of *mdx* Mice

Muscle degeneration in *mdx* mice is characterized by high oxidative stress [11]; therefore, we asked whether miR-200c was induced in *mdx* mice compared to *wt* mice.

**Figure 5:**
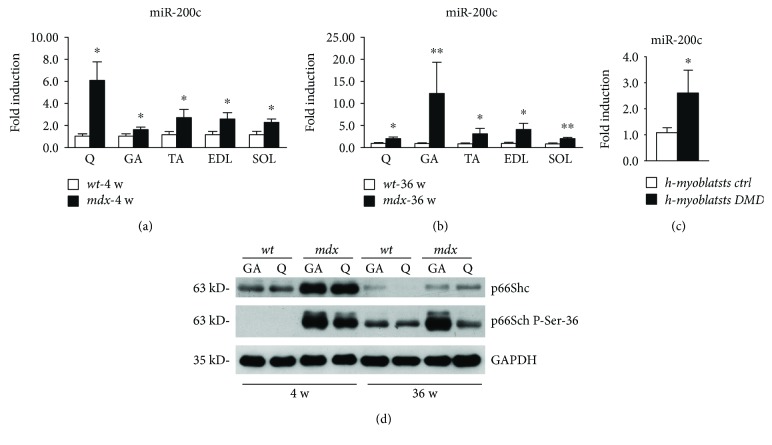
miR-200c and p66Shc phosphorylation in Ser-36 increase in dystrophic muscles of *mdx* mice. (a) The mRNA of quadriceps (Q), gastrocnemius (GA), tibialis anterior (TA), extensor digitorum longus (EDL), and soleus (SOL) muscles isolated from 3 young (4-week-old (4 w)) *mdx* mice were assayed for miR-200c expression. miR-200c increased in young *mdx* mice compared to young *wt* mice (*n* = 3 for each muscle; ^∗^*p* < 0.05; the bar graphs are average results of miR-200c expression levels of 3 different mice for each muscle). (b) The mRNA of quadriceps (Q), gastrocnemius (GA), tibialis anterior (TA), extensor digitorum longus (EDL), and soleus (SOL) muscles isolated from 3 old (36-week-old (36 w)) *mdx* mice were assayed for miR-200c expression. miR-200c increased in old *mdx* mice compared to old *wt* mice (*n* = 3 for each muscle; ^∗∗^*p* < 0.01; ^∗^*p* < 0.05; the bar graphs are average results of miR-200c expression levels of 3 different mice for each muscle). (c) The mRNA of human myoblasts derived from muscle biopsies of healthy donors or DMD patients and differentiated *in vitro* were assayed for miR-200c expression. miR-200c increased in DMD myoblasts compared to control myoblasts (*n* = 3; ^∗^*p* < 0.05; the bar graphs are average results of miR-200c expression levels of 3 different cell populations for control or DMD samples). (d) A representative Western blot of GA and Q protein extracts of both young (4 w) and old *mdx* (36 w) compared to young and old *wt* mice showed that p66 protein was induced in *mdx* mice compared to *wt* mice. Moreover, the phosphorylation in Ser-36 was induced in *mdx* mice compared to *wt* mice in both young and old mice (the experiment was performed on 3 biological replicates; 3 *wt* mice and 3 *mdx* mice of 4 weeks and 36 weeks, resp.). Glyceraldehyde-3-phosphate dehydrogenase (GAPDH) was used as loading control.

We analyzed miR-200c expression levels in different muscles, that is, quadriceps (Q), gastrocnemius (GA), tibialis anterior (TA), extensor digitorum longus (EDL), and soleus (SOL), in both young (4-week-old mice (4 w)) and older mice (36-week-old mice (36 w)) (Figures 5(a) and 5(b)). We found that miR-200c was significantly higher in *mdx* mice compared to *wt*, in all muscle groups examined, both in young and older mice (Figures 5(a) and 5(b)); indeed, in Q of young (~6-fold) and in GA of older mice (~12-fold), we found a very high increase of miR-200c expression (Figures 5(a) and 5(b)). An increase of miR-200c expression was also found in human myoblasts derived from muscle biopsies of DMD patients cultured in muscle differentiation medium, compared to human-differentiated myoblasts derived from muscle biopsies of healthy donors (Figure 5(c)).

In light of these results, we examined the levels of p66Shc phosphorylation in Ser-36 in Q and GA.

Interestingly, we found an increase of p66 protein in young *mdx* compared to *wt* mice and a strong upregulation of Ser-36 phosphorylation (Figure 5(d)). Notably, p66 phosphorylation in Ser-36 was increased in older mice also in basal conditions and was even higher in Q and particularly in GA, showing that older mice displayed higher miR-200c (Figure 5(d)).

These results suggest a role for miR-200c in oxidative stress increase in *mdx* mice, mediated, at least in part, by p66Shc-dependent mechanism.

## 4. Discussion

In this report, we dissected the role of the oxidative stress-induced miR-200c on muscle differentiation, since our and other laboratories reported a decrease in myogenic differentiation upon oxidative stress *in vitro* [1, 27–29].

Our results showed that miR-200c inhibits myogenic differentiation when forced miR-200c overexpression was performed in myoblasts; moreover, when miR-200c is overexpressed in differentiated myotubes, a decrease in myotube number and size was also observed. These effects are associated with a decrease in myogenic markers, that is, MyHC and myogenin, both in growing and in differentiating conditions. In keeping, anti-miR-200c treatment in growing myoblasts accelerates myogenic differentiation, increasing myotube numbers and size. We previously found that miR-200c induces oxidative stress and p66Shc phosphorylation in Ser-36 in endothelial cells [10]. In the present study, we confirmed these results also in murine myoblasts. Further, we found that both miR-200c and p66Shc phosphorylation in Ser-36 increase in *mdx* muscles compared to *wt*. Moreover, we found that miR-200c is significantly induced in human-differentiated myoblasts of DMD patients compared to differentiated myoblasts of healthy donors.

Other papers evaluated miRNA expression modulation in *mdx* and DMD muscles and also in sera [13, 30]. Indeed, Greco et al. found that in adductor muscles of *mdx*, a miR-200c upregulation was present, although not significant [13]. Moreover, the authors demonstrated that degenerative miRNAs may regulate, at least in part, critical mediators of cell death, contributing to the apoptotic/necrotic myofiber loss associated with both ischemia and DMD [13]. Our previous data show that miR-200c is highly induced by ischemia and its inhibition is able to increase limb perfusion, reverting the downregulation of its targets responsible of apoptosis, senescence, ROS increase, and nitric oxide (NO) decrease [10]. In keeping, the present study demonstrates that miR-200c that we previously showed to be upregulated upon ischemia [9, 10] is associated with myotube loss and is upregulated in *mdx* and DMD.

Our previous studies demonstrated that p66Shc^−/−^ SC differentiated better than *wt* cells in terms of myogenic marker increase, that is, myogenin and MyHC and myotube numbers, assessed by MyHC fluorescence; further, myogenic conversion, induced by MyoD overexpression, was more efficient in p66Shc^−/−^ fibroblasts compared to *wt* cells [1]. The explanation for this, was ascribed to lower oxidative stress in p66Shc^−/−^ cells compared to *wt* cells. In addition, it is possible that NO plays a positive role in higher and faster myogenic regeneration potential of p66Shc^−/−^ mice and cells. Indeed, NO mediates SC activation [31] and it is required for myoblast fusion [32]. Since ROS rapidly react with NO, generating nitrogen species, such as peroxynitrite [33], it is conceivable that p66Shc deletion enhances nitric oxide bioavailability [34], thus, favoring myogenic differentiation.

DMD is a genetic disease caused by deficiency of dystrophin, a critical component of the DGC, acting as a link between cytoskeleton and extracellular matrix in skeletal and cardiac muscles [15, 16]. A direct consequence of the DGC inefficiency is muscle fragility, contraction-induced damage, necrosis, reduced NO [35], oxidative stress, and inflammation.

In keeping, different preclinical studies in *mdx* mice report benefits, that is, decreased necrosis and improved muscle pathology, for many antioxidant drugs and interventions [11].

Resveratrol, among others, is an antioxidant drug that has a positive role on DMD [36]; interestingly, it is a potent activator of sirtuin1 (SIRT1). Moreover, SIRT1 overexpression in muscle reverses the phenotype of *mdx* mice [37].

SIRT1 is a NAD^+^-dependent deacetylase that displays antioxidant properties and enhances NO bioavailability [38].

Our recent report demonstrated that miR-200c targets directly SIRT1 and also two important proteins that modulate NO production and ROS scavenger transcription, that is, endothelial nitric oxide synthase (eNOS) and FOXO1. Therefore, we showed that miR-200c upregulation decreases NO, increases ROS production, and induces p66Shc protein phosphorylation in Ser-36; this, in turn, induces ROS via different mechanisms, one of which is the inhibition of FOXO1 transcription of ROS scavengers, reinforcing this molecular circuitry [10].

Taken together, these results suggest a pivotal role of miR-200c in oxidative stress induction in DMD via a p66Shc-dependent mechanism. miR-200c upregulation might contribute to the establishment of the negative consequences associated with this muscle disease, such as muscle wasting, lack of muscle regeneration, necrosis, NO decrease, and oxidative stress increase.

## 5. Conclusion

p66Shc phosphorylation in Ser-36 is increased in *mdx* muscles, and miR-200c expression levels are upregulated both in *mdx* muscles and in differentiated human myoblasts of DMD. Although further experiments should be accomplished in order to point to miR-200c as a therapeutic target, these data strongly suggest its possible involvement in muscle wasting in DMD, through apoptosis and senescence induction, as well as, by the induction of ROS and the decrease of NO.
